# COVID-19 in-patient hospital mortality by ethnicity

**DOI:** 10.12688/wellcomeopenres.15913.1

**Published:** 2020-05-04

**Authors:** Gillian Santorelli, Trevor Sheldon, Jane West, Chris Cartwright, John Wright

**Affiliations:** 1 Bradford Institute for Health Research, Bradford, BD9 6RJ, UK; 2 Department of Health Sciences, University of York, UK, York, YO10 5DD, UK

**Keywords:** Covid19, mortality, ethnicity, gender

## Abstract

There is debate about the extent to which COVID-19 affects ethnic groups differently. We explored if there was variation in hospital mortality in patients with COVID. Mortality rates in 1,276 inpatients in Bradford with test results for COVID-19 were analysed by ethnic group. The age-adjusted risk of dying from COVID-19 was slightly lower in South Asian compared to White British patients. (RR =0.87, 95% CI: 0.41 to 1.84).

## Introduction

There is a concern that COVID-19 may be disproportionately affecting people from ethnic minorities ^1^. This could be because of increased exposure, affecting incidence, underlying comorbidities and other factors affecting severity of disease and mortality following infection. There are few reliable data on community prevalence of infection. However, hospital electronic records contain information on ethnicity, test results and mortality. We aimed to explore variations in hospital mortality for people testing positive by ethnic group.

## Methods

### Data sources

Information on age, sex, ethnicity, COVID-19 test result and death were collected on all hospital patients who were tested for COVID-19 between 18 ^th^ March to 27 ^th^ April 2020 in a large teaching hospital in Bradford, a city with a diverse population of which 24.9% are of South Asian (20.4% Pakistani) origin ^2^. We compared the mortality rate of those with a positive and negative COVID-19 test result in hospital and explored whether this differential varied by gender, and ethnicity. These de-identified data are available from Harvard Dataverse ^3^.

### Statistical analysis

In order to take into account possible confounding due to factors related to age and the likelihood of hospitalisation or of being tested, we estimated the ratio of the risk of death in those tested positive relative to the risk in those also hospitalised, but who tested negative with the same gender or belonging to the same ethnic group, adjusted for age using multivariable regression. Mortality rates of White British and South Asian patients were compared using a chi-square test. All analyses were conducted using STATA/SE software (Stata/SE 15, StataCorp, College Station, TX, USA).

### Ethical considerations

The recent Health Service (Control of Patient Information) Regulations 2002 notices requires NHS Trusts and others to process confidential patient information without consent for COVID-19 public health, surveillance and research purposes, thus no ethical approval or consent for publication was required.

## Results

In total, 812 patients tested negative (63.6%) and 464 positive (36.4%). The overall mortality rate in those testing positive for COVID-19 was 23.5% (see Table 1). This was over twice the mortality rate of those inpatients with negative results (8.9%), risk ratio (RR) = 2.65 (95% confidence interval (CI): 2.02 to 3.49).

**Table 1.** Characteristics of the hospital inpatients tested for COVID-19. Numbers are n (%) except where otherwise stated.

Characteristic	Tested	Test for COVID-19		Died	
	n=1,276	Negative n=812 (63.6)	Positive n=464 (36.4)	Negative n=72 (8.9)	Positive n=109 (23.5)
Age, years, median	65	62	68	80.5	76
Sex					
Male	661	403 (61.0)	258 (39.0)	39 (36.5)	68 (63.6)
Female	615	409 (66.5)	206 (33.5)	33 (44.6)	41 (55.4)
Ethnic group					
White: British	656	439 (66.9)	217 (33.1)	44 (44.4)	55 (55.6)
South Asian	322	195 (60.6)	127 (39.4)	16 (41.0)	23 (59.0)
Other	187	108 (57.8)	79 (42.3)	7 (25.0)	21 (75.0)
Not stated	111	70 (63.1)	41 (36.9)	5 (33.3)	10 (66.7)

The mortality rate in those testing positive for COVID-19 was higher in White British patients (25.4%) than those of South Asian origin (18.1%) but this was not statistically significant (P-value 0.122). Mortality among non-COVID-19 cases was 10.0% and 8.2% in White British and South Asian patients, respectively.

South Asian origin patients were significantly younger than White British patients (mean 49 vs 66 years), reflecting the local demographics. The age-adjusted RR of dying after a positive versus a negative result was 1.98 for women, 2.11 for men, 2.10 for White British and 1.72 for South Asian. The age-adjusted relative increased risk of dying from COVID-19 compared to test negative was lower, though not statistically significantly so, in South Asian compared to White British (RR = 0.87; 95% CI 0.41 to 1.84).

## Conclusions

Data from the last month of the outbreak in a large District general hospital in Bradford show that COVID-19 increases risk of death for infected individuals compared to hospital patients with similar symptoms with no COVID-19 infection. They also suggest that this increased risk is not greater in people of South Asian (mainly Pakistani) ethnicity.

These data are only for hospitalised patients, and do not account for patients who subsequently died after the date of analysis. It also sheds no light on variations by ethnicity in exposure to, risk of coronavirus infection or the severity of COVID-19 illness in the community.

## Data availability

### Underlying data

Harvard Dataverse: Replication Data for COVID-19 in-patient hospital mortality by ethnicity. https://doi.org/10.7910/DVN/l6ORLP ^3^.

‘Covid19EthinicityData’ contains de-identified patient-level data analysed in this study.

Data are available under the terms of the Creative Commons Zero ‘No rights reserved’ data waiver (CC0 1.0 Public domain dedication).
