# Supplementary material for: COVID-19 in-patient hospital mortality by ethnicity
Source: Wellcome Open Res. 2020 May 4;5:86. [Version 1] doi: 10.12688/wellcomeopenres.15913.1 (PMC7308809; doi:10.12688/wellcomeopenres.15913.1)
Supplement: Peer review history

# Peer review: Reviewer response for version 1

**DOI:** 10.21956/wellcomeopenres.17454.r38762

**Published:** 2020-06-15

**Authors:** Arpana Verma

**Affiliations:** 1 Manchester Academic Health Sciences Centre, School of Health Sciences, University of Manchester, Manchester, M13 9PT, UK

Many thanks for a well researched and timely piece of analysis from a large teaching hospital with a large population from BAME communities (N.B. South Asian and Pakistani in origin).

 The dataset can be downloaded from the Harvard Dataverse and includes all the participants listed in the paper to allow for replication of results.

 I agree with the conclusions and would only ask for the abstract to reflect this - perhaps "The age-adjusted risk of dying from COVID-19 was not significant but slightly lower in South Asian compared to White British patients. (RR =0.87, 95% CI: 0.41 to 1.84)."

 Otherwise an excellent paper that will add to the evidence base and perhaps this publication will facilitate further consortia combing datasets coming together to improve the evidence base further.

Is the work clearly and accurately presented and does it cite the current literature?

Yes

If applicable, is the statistical analysis and its interpretation appropriate?

Yes

Are all the source data underlying the results available to ensure full reproducibility?

Yes

Is the study design appropriate and is the work technically sound?

Yes

Are the conclusions drawn adequately supported by the results?

Partly

Are sufficient details of methods and analysis provided to allow replication by others?

Yes

Reviewer Expertise:

Public health and epidemiology.

I confirm that I have read this submission and believe that I have an appropriate level of expertise to confirm that it is of an acceptable scientific standard.

# Peer review: Reviewer response for version 1

**DOI:** 10.21956/wellcomeopenres.17454.r38627

**Published:** 2020-06-01

**Authors:** Colin Simpson

**Affiliations:** 1 School of Health, Faculty of Health, Victoria University of Wellington, Wellington, New Zealand

This is an interesting and clearly presented study which used hospital data from a large Teaching Hospital in Bradford to explore variations in hospital mortality for people testing positive for SARS-CoV-2 by ethnic group. De-identified data was sourced from the Harvard Dataverse.

 Mortality for those with positive and negative test results in hospital were explored using a multivariate regression analysis for any variation in outcome by gender and ethnicity, adjusting for a number of confounding factors. The methodological approach seems an appropriate analysis for this study.

 Amongst other study findings, the study found that mortality for COVID-19 for patients of South Asian (mainly Pakistani) ethnicity was lower (although not significantly) than White British ethnicity.

 It is important to clarify throughout that tests were performed on symptomatic patients (and presumably not all patients) i.e. there were testing criteria. Where described in the report, the comparison group for this project should be stated as ‘hospital patients (with similar symptoms to positive) tested negative’ or similar.

 In the study conclusions, further cautious language could be used, for instance there could be a brief discussion regarding the power of the study to detect differences in mortality by sex and ethnicity.

Is the work clearly and accurately presented and does it cite the current literature?

Yes

If applicable, is the statistical analysis and its interpretation appropriate?

Yes

Are all the source data underlying the results available to ensure full reproducibility?

Yes

Is the study design appropriate and is the work technically sound?

Yes

Are the conclusions drawn adequately supported by the results?

Yes

Are sufficient details of methods and analysis provided to allow replication by others?

Yes

Reviewer Expertise:

Infectious Disease Epidemiology.

I confirm that I have read this submission and believe that I have an appropriate level of expertise to confirm that it is of an acceptable scientific standard.
